# Effect of Tween Series on Growth and *cis*-9, *trans*-11 Conjugated Linoleic Acid Production of *Lactobacillus acidophilus* F0221 in the Presence of Bile Salts

**DOI:** 10.3390/ijms12129138

**Published:** 2011-12-08

**Authors:** Jing-Yan Li, Lan-Wei Zhang, Ming Du, Xue Han, Hua-Xi Yi, Chun-Feng Guo, Ying-Chun Zhang, Xue Luo, Yan-He Zhang, Yu-Juan Shan, Ai-Ju Hou

**Affiliations:** 1 School of Food Science and Engineering, Harbin Institute of Technology, Harbin 150090, China; E-Mails: jyli@hit.edu.cn (J.-Y.L.); duming121@163.com (M.D.); jiayuanhan@yahoo.com.cn (X.H.); yihx2006@yahoo.com.cn (H.-X.Y.); zyc229@163.com (Y.-C.Z.); luoxue1984@126.com (X.L.); yujuan72@163.com (Y.-J.S.); aijuhellea@yahoo.com.cn (A.-J.H.); 2 School of Food Science and Engineering, Northwest A & F University, Yangling 712100, China; E-Mail: gcf@nwsuaf.edu.cn; 3 National Key Laboratory of Veterinary Biotechnology, Harbin Veterinary Research Institute, Chinese Academy of Agricultural Sciences, Harbin 150001, China; E-Mail: yhz7606@yahoo.com.cn

**Keywords:** *cis*-9, *trans*-11 conjugated linoleic acid, *Lactobacillus acidophilus* F0221, bile salts, Tween 80, permeability

## Abstract

*Cis-9*, *trans*-11 conjugated linoleic acid (*c*9, *t*11 CLA) producing bacteria have attracted much attention as novel probiotics which have shown beneficial effects on host health. However, bile salts are able to inhibit bacterial growth and *c*9, *t*11 CLA production. For recovering growth and *c*9, *t*11 CLA production of *Lactobacillus acidophilus* F0221 in the presence of bile salts, Tween series (Tween 20, Tween 40, Tween 60 and Tween 80) were added in growth culture containing 0.3% oxgall. Results showed that the viable counts were significantly (*P* < 0.05) recovered to 8.58–8.75 log CFU/mL in the presence of all Tween treatments. However, recovery of *c*9, *t*11 CLA production was only demonstrated in the presence of Tween 80 (72.89 μg/mL). Stepwise increasing oxgall in a concentrations range from 0.1% to 0.9% according to human intestinal physiological environments, Tween 80 still showed significant (*P* < 0.05) recovery ability on growth (8.91–8.04 log CFU/mL) and *c*9, *t*11 CLA (69.22–34.27 μg/mL) production. The effect of Tween 80 on growth and production was also investigated in the presence of different types of bile salts (sodium salts of cholic acid (CA), deoxycholic acid (DCA), chendeoxycholic acid (CDCA), glycocholic acid (GCA) and taurocholic acid (TCA)). Results showed that Tween 80 could significantly (*P* < 0.05) recover *c*9, *t*11 CLA production in the presence of all types of bile salts, but the Tween 80 could only significantly (*P* < 0.05) recover viable counts of the strain in the presence of CA, DCA and CDCA. This recovery ability could be attributed to the protection of leakage of intracellular material. Additionally, although bile salts inhibited growth and *c*9, *t*11 CLA production by the growing cell, it promoted the *c*9, *t*11 CLA production by the resting cell.

## 1. Introduction

Conjugated linoleic acid (CLA) represents a group of positional and geometric isomers of octadecadienoic acids with conjugated double bonds [1]. It has exhibited important physiological activities, such as having anticarcinogenic activity [2], enhancing cell immunity [3], reducing body fat content [4], inhibiting arteriosclerosis [5] and modulating the blood-glucose and insulin tolerance [6]. These beneficial activities are mainly attributed to the *cis-*9, *trans-*11 CLA (*c*9, *t*11 CLA). The CLA cannot be synthesized in the human body [7,8], and the presence of *c*9, *t*11 CLA in human tissue (adipose, plasma and intestine) derives from food intake [9].

The main natural sources of *c*9, *t*11 CLA are meat and milk of ruminants, and the content depends on the kind of ruminants, feeding seasons and conditions [10]. Despite the higher content in ruminant products, ingestion of *c*9, *t*11 CLA by this manner is recognized as an impracticable approach to promote human health [2,11,12]. Currently, commercial *c*9, *t*11 CLA supplements are mainly synthesized by alkaline isomerization of vegetable oil, whereas the disadvantages of the alkaline isomerization are that the processes often do not produce a single *c*9, *t*11 CLA isomer at high purity [13]. Another better alternative is to convert LA to the *c*9, *t*11 CLA by bacteria in the gastrointestinal tract. Several studies have demonstrated that intake of the CLA producing bacteria such as *L. rhamnosus* PL60 [14], *L. rhamnosus* PL62 [15], and *Bifidobacterium breve* NCIMB 702258 [16] could improve the levels of *c*9, *t*11 CLA in intestinal lumen. However, bile salts as major antimicrobial components inhibit growth of bacteria by disruption of the permeability and integrity of the cell membrane [17,18].

Certain substances are able to protect live bacterial cells against bile salts toxicity. These antagonists include carbohydrates, surfactants and free amino acids [19,20]. They can form different complexes with the salts, and thus inhibit the toxicity to live bacterial cells. According to the report of Kimoto *et al*. [21], Tween series exhibited an apparent recovery ability on the growth of lactococci in the presence of bile salts. However, according to our best knowledge, the effect of Tween series on growth and *c*9, *t*11 CLA production in the presence of bile salts have not previously been reported. Therefore, the aim of this study was to investigate the bile salts toxicity on *L. acidophilus* F0221 growth and production of *c*9, *t*11 CLA and also determine the activity of Tween series on diminishing of bile salts toxicity.

## 2. Results

### 2.1. Effect of Oxgall on Growth and c9, t11 CLA Production

**Figure 1:**
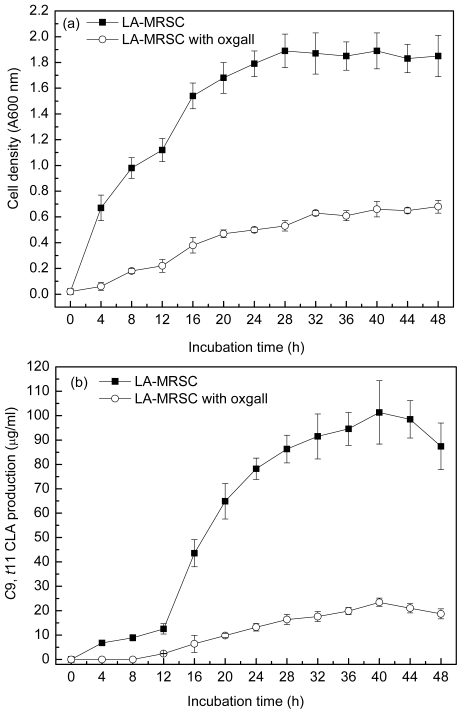
Time curves of growth (**a**) and *c*9, *t*11 CLA production (**b**) in LA-MRSC broth in the presence and absence of oxgall.

Figure 1a and 1b showed the ability to grow and produce *c*9, *t*11 CLA in the absence and presence of 0.3% oxgall in the LA-MRSC (MRS broth supplemented with 0.5 g/L LA and 0.5 g/L cystein-HCl) broth for the 48 h incubation period, respectively. During the incubation period, a tremendous increase in the absorbance value was observed in the medium without oxgall addition. The maximal cell density reached approximately 1.89 (A600 nm) after 28 h incubation. *C*9, *t*11 CLA production was positively correlated with the cell density. The production was the highest (101.32 μg/mL) at the middle of stationary growth phase (40 h), whereas there was a slight decrease at the end of the stationary growth phase (48 h). When the LA-MRSC broth was supplemented with 0.3% oxgall, no detectable accumulation of *c*9, *t*11 CLA was observed in the early phase of growth (0–12 h). At the stationary growth phase (40 h), oxgall toxicity caused significant decrease of *c*9, *t*11 CLA production (23.45 μg/mL), which was nearly fivefold lower than in oxgall absence.

### 2.2. Effect of Tween Series on Growth and c9, t11 CLA Production in the Presence of Oxgall

**Figure 2:**
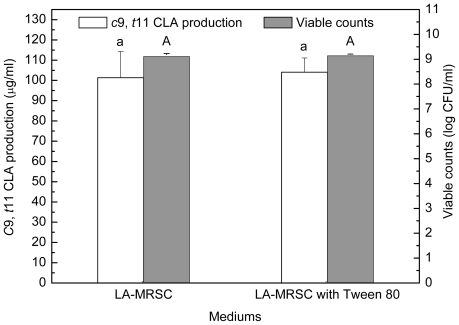
Effect of Tween series on the growth and *c*9, *t*11 CLA production in the presence of 0.3% oxgall. Means with different lowercase letters (a and b) differ significantly (*P* < 0.05) in the viable counts. Means with different uppercase letters (A and B) differ significantly (*P* < 0.05) in the *c*9, *t*11 CLA production.

**Figure 3:**
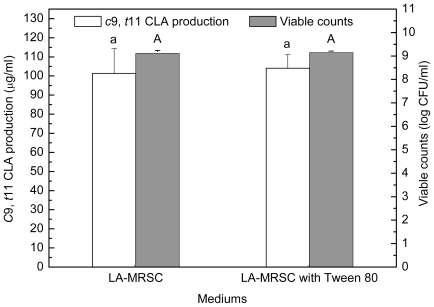
Effect of Tween 80 on the growth and *c*9, *t*11 CLA production in LA-MRS broth. Means with same lowercase letters does not differ significantly (*P* > 0.05) in the viable counts. Means with same uppercase letters does not differ significantly (*P* > 0.05) in the *c*9, *t*11 CLA production.

Compared to those grown in LA-MRSCO broth (LA-MRSC broth supplemented with 0.3% oxgall), viable counts were significantly (*P* < 0.05) recovered to 8.58–8.75 log CFU/mL from 7.99 log CFU/mL in the absence of Tween by the four kinds of Tween (Figure 2). As for *c*9, *t*11 CLA production, the results indicated that only Tween 80 showed recovery ability. The production was significantly (*P* < 0.05) recovered from 22.34 μg/mL in the absence of Tween 80 to 72.89 μg/mL in the presence of the Tween 80. Although Tween 20, Tween 40 and Tween 60 were also observed to be effective in recovering cell growth, they were not able to recover the *c*9, *t*11 CLA production. Additionally, for excluding the effect of Tween 80 on *c*9, *t*11 CLA production in the absence of oxgall, cell density and *c*9, *t*11 CLA production were determined in LA-MRSC broth with or without Tween 80. The results showed that Tween 80 did not significantly (*P* < 0.05) affect growth and *c*9, *t*11 CLA production (Figure 3).

### 2.3. Effect of Tween 80 on Growth and c9, t11 CLA Production in the Presence of Different Concentrations of Oxgall

**Figure 4:**
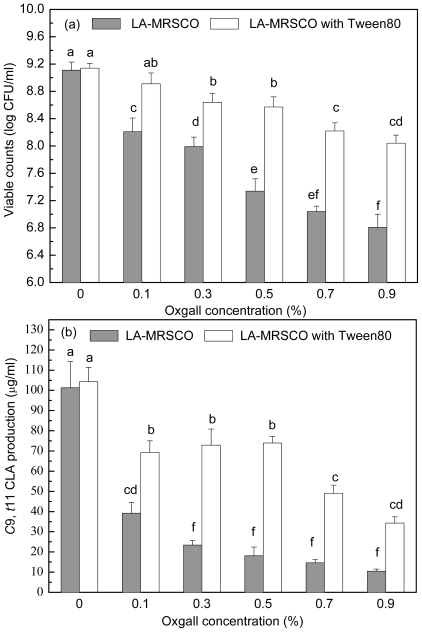
Effect of Tween 80 on growth (**a**) and *c*9, *t*11 CLA production (**b**) in the presence of different concentrations of oxgall. Values not sharing the same superscript are significantly different (*P* < 0.05).

Stepwise increasing oxgall of concentrations ranged from 0% to 0.5% led to a gradual decrease in both viable count (from 9.11 to 7.34 log CFU/mL) and *c*9, *t*11 CLA production (from 101.32 to 18.09 μg/mL, Figure 4a,b). Further increases of oxgall of concentrations to 0.7% and 0.9% resulted in death of the inoculated cells (7.04 and 6.81 log CFU/mL). However, a small amount *c*9, *t*11 CLA (14.61 and 10.43 μg/mL) was produced in the presence of 0.7% and 0.9% oxgall.

When Tween 80 was added to the different concentrations of oxgall (0.1%–0.9%) LA-MRSC broths, viable counts and *c*9, *t*11 CLA productions were significantly (*P* < 0.05) recovered to 8.91–8.04 log CFU/mL and 69.22–34.27 μg/mL, respectively. In addition, the ability of recovery of Tween 80 on viable counts and *c*9, *t*11 CLA production in 0.1%–0.5% oxgall LA-MRSC broth was significantly (*P* < 0.05) higher than the counts and production in the broth supplemented with 0.7 and 0.9% oxgall.

### 2.4. Effect of Tween 80 on Growth and c9, t11 CLA Production in the Presence of Different Types of Individual Bile Salts

**Figure 5:**
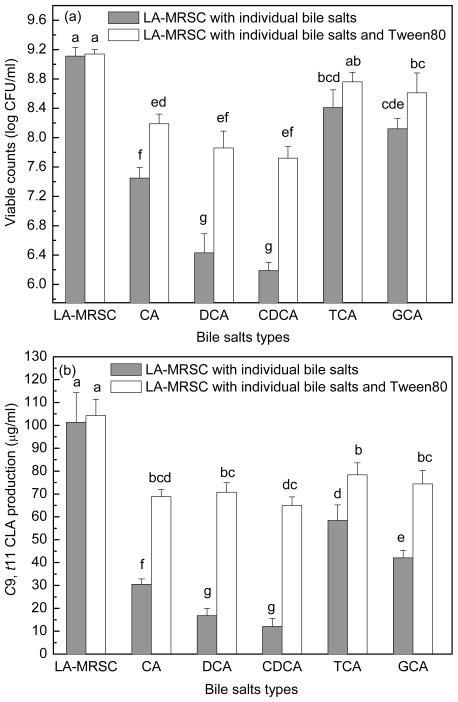
Effect of Tween 80 on growth (**a**) and *c*9, *t*11 CLA production (**b**) in the presence of different types of individual bile salts. Values not sharing the same superscript are significantly different (*P* < 0.05).

Figure 5a,b showed the antibacterial activity of individual bile salts against growth and *c*9, *t*11 CLA production. When around 7.11 log CFU/mL cells were inoculated in TCA and GCA-LA-MRSC broths, viable counts increased to 8.41 and 8.12 log CFU/mL, respectively. In CA-LA-MRSC broth, the cells showed little growth (7.45 log CFU/mL), and in the broth containing other types of individual bile salts (DCA and CDCA), some of the inoculated cells died (6.43 and 6.19 log CFU/mL). As for the *c*9, *t*11 CLA production, it was decreased to 58.43 and 42.10 μg/mL by conjugated bile salts TCA and GCA, and to 30.55–12.05 μg/mL by deconjugated bile salts CA, DCA and CDCA.

When Tween 80 was added to the CA, DCA and CDCA-LA-MRSC broths, viable counts were significantly (*P* < 0.05) recovered to 8.19, 7.86 and 7.72 log CFU/mL, respectively. Tween 80 did not significantly (*P* < 0.05) recovered bacteria growth in TCA and GCA-LA-MRSC broths. When compared with *c*9, *t*11 CLA production, the Tween 80 significantly (*P* < 0.05) recovered the production to 78.33 and 74.41 μg/mL in the presence of conjugated bile (TCA and GCA) and to 68.85–70.74 μg/mL in the presence of deconjugated bile salts (CA, DCA and CDCA), respectively.

### 2.5. Effect of Tween 80 on Leakage of Cellular Material in the Presence of Bile Salts by Resting Cell

**Figure 6:**
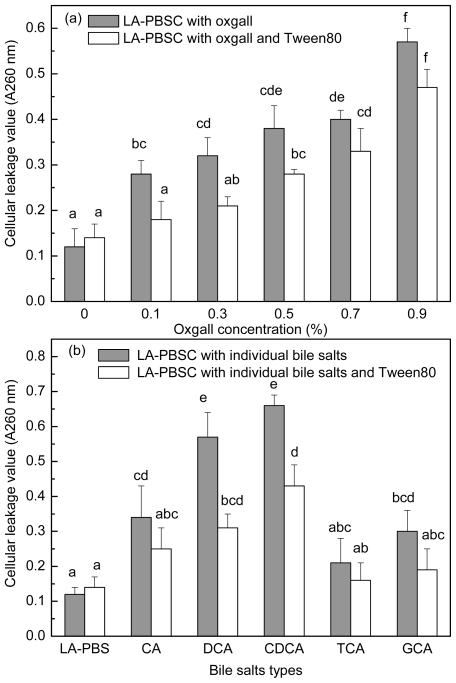
Effect of Tween 80 on leakage of intracellular material in the presence of different concentrations (**a**) and types of individual bile salts (**b**).Values not sharing the same superscript are significantly different (*P* < 0.05).

The stationary phase cells were harvested and inoculated into LA-PBSC (phosphate buffer saline (PBS) supplemented with 0.5 g/L LA and 5 g/L cystein-HCl) containing different concentrations of oxgall and different types of individual bile salts with or without Tween 80. As shown in Figure 6a,b, 0.1%–0.9% oxgall and 0.3% CA, DCA, CDCA and GCA resulted in leakage of intracellular material of the cell. In LA-PBSC containing 0.1%–0.3% oxgall and 0.3% DCA and CDCA, Tween 80 significantly (*P* < 0.05) decreased the degree of leakage of intracellular material, however, in the presence of 0.5%–0.9% oxgall and 0.3% CA, TCA and GCA, Tween 80 did not have a significant (*P* > 0.05) effect.

### 2.6. Effect of Bile Salts on c9, t11 CLA Production by Resting Cell

**Figure 7:**
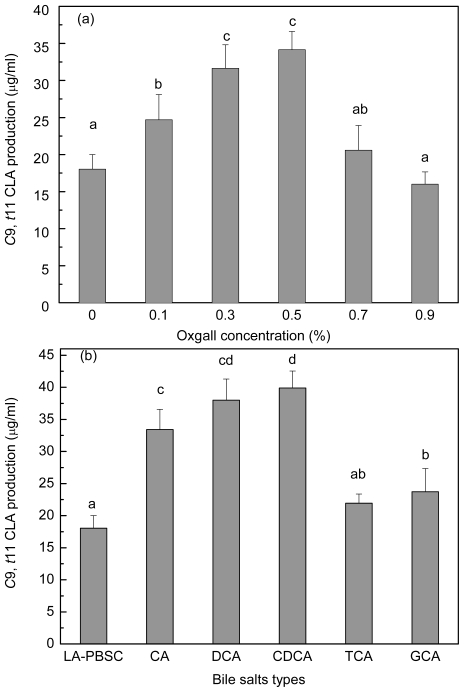
Effect of different concentrations (**a**) and types (**b**) of individual bile salts on *c*9, *t*11 CLA production of resting cell. Values not sharing the same superscript are significantly different (*P* < 0.05).

In order to investigate the bile salts effect on *c*9, *t*11 CLA production of resting cell, stationary-phase cells were harvested and incubated into the LA-PBSC containing different concentrations of oxgall (0.1%–0.9%) and 0.3% different types of individual bile salts (Figure 7a,b). Results showed that the *c*9, *t*11 CLA production was significantly (*P* < 0.05) enhanced in the presence of 0.1%–0.5% oxgall compared with the control. The production reached maximum with 0.5% oxgall (34.15 μg/mL), which showed an approximately two-fold higher level than the control (18.03 μg/mL). Further increase of oxgall of concentrations to 0.7% and 0.9% did not show significantly (*P* > 0.05) increase in *c*9, *t*11 CLA production.

Different types of individual bile salts also exhibited different degrees of effect on *c*9, *t*11 CLA production. Compared with the control (18.03 μg/mL), conjugated bile salt TCA did not significantly (*P* > 0.05) affect *c*9, *t*11 CLA production (21.93 μg/mL). However, other bile salts (GCA, CA, DCA and CDCA) significantly (*P* < 0.05) enhanced the production to 23.73 μg/mL, 33.42, 37.98 and 39.91 μg/mL, respectively.

## 3. Discussion

Since bile salts are surfactant-like compounds with recognized strong antimicrobial activity, bile salts tolerance is usually considered as a prerequisite to evaluate bacteria propagation and beneficial function in the intestine. In the absence of oxgall, the maximal cell density of the strain reached 1.89 (A600 nm) after 28 h incubation and the maximal *c*9, *t*11 CLA production reached 101.32 μg/mL at stationary growth. Because bacteria passing through the small intestine might be in a physiological state similar to the stationary phase [22], we speculated that the strain could be used as a probiotic which can exert biological activity in the human intestine.

Compared with the growth in the medium without oxgall, the absorbance of the culture had reached 0.68 ± 0.05 when strain was grown in the medium with 0.3% oxgall, indicating that bile salts had a significant inhibitory action on growth of the strain. Because of the strong growth inhibitory action, only very small amounts of LA could be converted to *c*9, *t*11 CLA.

Although bile salts have been identified to have antimicrobial toxicity, the toxicity is reversible. In many instances the reversibility is modulated by the presence of certain substances such as carbon and nitrogen sources and surface-active substances [19,20]. In the present study, the use of Tween series is also effective for this objective. The order of growth recovery ability was Tween 80 > Tween 60 > Tween 40 > Tween 20, and was probably due to the difference in molecular structures and hydrophilic-lipophilic balance (HLB) values of the four kinds of Tween solutions [23]. The anti-inhibitory effects of Tween series might be rationalized in terms of an ability to form micelles complexes with bile salts, thereby facilitating the removal of bile salts from intimate contact with the bacterial cell surfaces [21].

However, as for *c*9, *t*11 CLA production, only Tween 80 showed a desirable effect. This effect was probably attributed to the specific molecular structure of Tween 80. Tween 80 contains oleic acid, which could be incorporated into bacteria cell membrane and further converted to cyclopropane fatty acid [24]. Jacques [25] has indicated that the cyclopropane fatty acid was one of the most important factors affecting cell membrane fluidity. The LA isomerase anchored to the cell membrane is responsible for the conversion of LA to *c*9, *t*11 CLA [26]. Consequently, it is likely that the increasing cell membrane fluidity results in supporting the impact of Tween 80 on the activity of LA isomerase.

Bile salts are widely distributed in the upper gastrointestinal tract and the concentration in the human intestine varies over time and with the different segments of the intestine [19,27]. In the duodenum, bile salts concentration reached approximately 0.75%, and in the ileum, the concentration decreased to approximately 0.2%. Bile salts at lower concentrations may disrupt membrane integrity and permeability, and at higher concentrations, the salts have induced the leakage of intracellular material and lysis [17]. In this study, the inhibition of growth and *c*9, *t*11 CLA production induced by 0.1%–0.5% oxgall was revovered by Tween 80. This result indicated that Tween 80 could promote the conversion of LA to *c*9, *t*11 CLA in the ileum.

Both conjugated and deconjugated bile salts were known to inhibit the growth of intestinal bacteria, especially gram-positive bacteria [17]. In this study, the deconjugated bile salts were more toxic than the conjugated bile salts. This result was in agreement with the report of Noriega *et al*. [28], who interpreted that the difference in the dissociation constant of different types of bile salts might be one of the major causes. Additionally, Kurdi *et al*. [29] and Suskovic *et al*. [30] indicated that deconjugated bile salt is a hydrophobic weaker acid salt and has a higher p*K*_a_ value than does the conjugated bile salt. Therefore, the relative amount of the protonated form is considerable. The protonated form of bile salts causes intracellular acidification and collapse of the proton motive force, which in turn results in inhibition of nutrient transport.

Presently, the precise mechanism for the antimicrobial activity of bile salts remains unclear. According to Taranto *et al*. [31], the change of chemical and physical properties of the cell membrane may account for the inhibitory activity. One of the means is by affecting the cellular permeability [21]. To investigate whether the permeability is a significant factor in recovery of cell growth and *c*9, *t*11 CLA production by Tween 80, the absorbing values at 260 nm were determined in LA-PBSC containing different concentrations and types of bile salts with or without Tween 80 addition. The results showed that 0.1%–0.9% oxgall and 0.3% CA, DCA, CDCA and GCA significantly (*P* < 0.05) increased cellular permeability. In the presence of 0.1%–0.3% oxgall and 0.3% DCA and CDCA, Tween 80 significantly (*P* < 0.05) reduced leakage of intracellular material, but in the presence of higher concentrations (0.5%–0.9%) oxgall, it showed no effect.

Additionally, although the ability of Tween 80 to inhibit toxicity of different types of individual bile salts on cell growth appeared significantly (*P* > 0.05) different, the recovery of *c*9, *t*11 CLA production was nearly consistent. The result is inconsistent with the report of Kim *et al*. [26], who indicated that *c*9, *t*11 CLA production was highly related to the viable counts. This leads us to speculate whether bile salt affect the *c*9, *t*11 CLA production on the resting cell. Therefore, we determined the effect of bile salts on *c*9, *t*11 CLA production by the resting cell. The result was consistent with our speculation that bile salts play a positive effect on *c*9, *t*11 CLA production. However, this phenomenon was only suitable for the resting cell, but not for the growing cell, since the toxicity of bile salts inhibited cell growth in growth medium and therefore decreased the amounts of resting cell.

It has been reported that bile salts can enhance the activity of certain endoenzymes such *β*-galactosidase and *β*-glucuronidase by increasing cell membrane permeability [32,33]. Additionally, Sanchez *et al*. [34] reported that bile salts could result in folds of the bacterial membrane protein, and then alter conformation of proteins. Linoleic acid isomerase is a membrane-bound enzyme, whose activity might be alterated by these approaches.

## 4. Experimental Section

### 4.1. Strain Isolation and Maintenance

Freshly collected human fecal sample was diluted 10-fold in anaerobic buffered peptone water containing 0.5 g/L cystein-HCl (Sigma Chemical Co., St. Louis, MO, USA), and spread on MRS agar plates [35] supplemented with 0.5 g/L cystein-HCl. The plates were incubated at 37 °C for 48 h in an anaerobic incubation system (model 1029, Forma Scientific Inc., Marietta, OH, USA) with an atmosphere of 85% N_2_, 10% H_2_ and 5% CO_2_. Pure colonies were collected and a *c*9, *t*11 CLA producing strain was screened out according to the method described by Chung *et al*. [36]. The strain was identified as *L. acidophilus* F0221 based on carbohydrate fermentation patterns by using API 50 CHL test kit (Biomérieux, Marcy-l’Etoile, France) with a computer-aided identification program (version 4.0, Biomérieux). The strain was maintained and sub-cultured in MRSC broth. Prior to assay, the strain was serially transferred three times at 37 °C for 18 h.

### 4.2. Bacteria Enumeration and Cell Density Measurement

The numbers of *L. acidophilus* F0221 in the experimental samples were determined using the spread plate method. The culture was serially diluted in 0.5% NaCl solution, appropriate dilutions were spread on MRSC agar plates incubated at 37 °C for 48 h under anaerobic conditions. Viable counts were described as log CFU/mL. Cell density was monitored by reading the absorbance at 600 nm (A600 nm) using a spectrophotometer (Ultrospec 1100 pro, Amersham Biosciences, UK).

### 4.3. Fatty Acid Analysis

The culture was inoculated into LA-MRSC broth and incubated at 37 °C for 48 h under anaerobic conditions. Fatty acid in culture (1 mL) was extracted with 6 mL of chloroform: methanol (2:1, v/v). Followed by 30 s vigorously shaking, the mixture was centrifuged (5000 g, 10 min, 4 °C). The chloroform phase was dried under a nitrogen flow in a water bath at 40 °C. The lipid residue was immediately methylated with 1 mL of 1 M sulfuric acid in methanol at 60 °C for 30 min. After cooling, the methylated sample was mixed with 1 mL *n*-hexane, shaken for 30 s, and was then centrifuged (5000 g, 5 min, 4 °C). The *n*-hexane layer was dehydrated with anhydrous sodium sulfate and analyzed for the *c*9, *t*11 CLA isomers.

**Figure 8:**
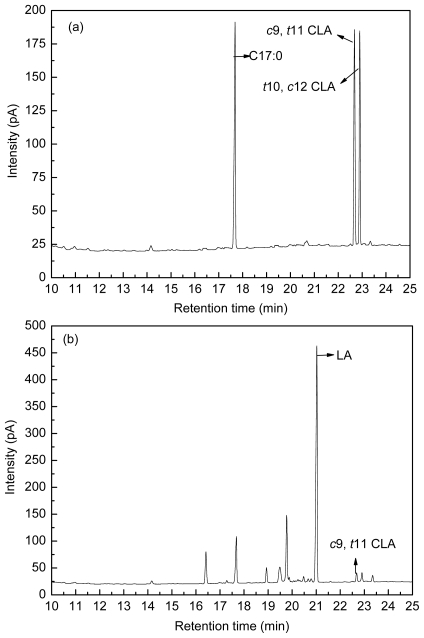

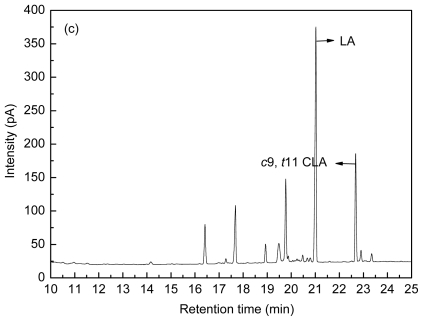
GC chromatogram for the fatty acid compositions. (**a**) C17:0, *c*9, *t*11 CLA and *t*10, *c*12 CLA standards; (**b**) LA-MRSC broth; (**c**) incubation of *L. acidophilus* F0221 in LA-MRSC broth.

The *c*9, *t*11 CLA isomers was analyzed using an Agilent 7890 gas chromatograph equipped with a FID detector and a fused silica capillary column HP-88 (100 m × 0.25 mm i.d., 0.2 μm film thickness, Agilent Technologies, Santa Clara, CA, USA). Heptadecanoic acid was added as the internal standard prior to the fatty acid extraction to determine the recovery rate. *c*9, *t*11 CLA was used as standard to identify and quantify the *c*9, *t*11 CLA isomer by comparison with the retention time and peak areas, and the amount in each sample was expressed as μg/mL. Figure 8 shows the fatty acid peak of C17:0, *c*9, *t*11 CLA and *t*10, *c*12 CLA standards (a), LA-MRSC broth (b) and *c*9, *t*11 CLA produced by *L. acidophilus* F0221 in LA-MRSC broth (c).

### 4.4. Cell Growth and c9, t11 CLA Production in the Presence of Oxgall

The overnight culture (1%) was inoculated into LA-MRSC broth supplemented with 0.3% oxgall and incubated under anaerobic conditions at 37 °C. Cell density and *c*9, *t*11 CLA production were monitored during the incubation period of 48 h. LA-MRSC broth without oxgall was used as a control.

### 4.5. Effect of Tween Series on Cell Growth and c9, t11 CLA Production in the Presence of Oxgall

The overnight culture (1%) was inoculated into LA-MRSCO broth supplemented with 1.5% (w/v) different Tween (Tween 20, Tween 40, Tween 60 and Tween 80). The culture was incubated under anaerobic conditions at 37 °C for 40 h, viable counts and *c*9, *t*11 CLA production were determined after incubation. LA-MRSCO broth without Tween was used as a control.

### 4.6. Effect of Tween 80 on Cell Growth and c9, t11 CLA Production in the Presence of Different Concentrations and Types of Individual Bile Salts

The overnight culture (1%) was inoculated into LA-MRSC broth supplemented 1.5% (w/v) Tween 80, different concentrations of oxgall (0%, 0.1%, 0.3%, 0.5%, 0.7% and 0.9% (w/v)) and 0.3% (w/v) different types of individual bile salts (CA, DCA, CDCA, GCA and TCA), respectively. The culture was incubated at 37 °C under anaerobic conditions. After 40-h incubation, viable counts and *c*9, *t*11 CLA productions were determined. In each experiment, the medium without Tween 80 was used as control.

### 4.7. Effect of Tween 80 on Leakage of Intracellular Material in the Presence of Bile Salts by Resting Cell

Leakage of intracellular material of the strain was determined according to the method of Kimeto *et al*. [21]. The overnight culture (0.5 mL) was inoculated into 50 mL LA-MRSC broth and incubated at 37 °C for 40 h under anaerobic conditions. Resting cell was harvested by centrifugation (12,000 g, 15 min, 4 °C), washed twice with phosphate buffer saline (PBS), and suspended in 10 mL of LA-PBSC supplemented with Tween 80 and different concentrations of oxgall (0.1%, 0.3%, 0.5%, 0.7% and 0.9%, w/v) or 0.3% (w/v) concentration of different types of individual bile salts (CA, DCA, CDCA, TCA and GCA). The media with Tween 80 was used to evaluate the effect of Tween 80 on leakage of intracellular material of the strain in the presence of bile salts, and the media without Tween 80 was used as control.

The culture was placed in a shaking water bath and incubated at 37 °C for 2 h. The suspensions were centrifuged (12,000 g for 15 min at 4 °C) and the leakage of cellular material of the cell was determined by measuring the supernatants as absorbance at 260 nm (A260 nm) with a Spectronic 20 spectrophotometer (Bausch and Lomb, Rochester, NY, USA) against a control.

### 4.8. Effect of Bile Salts on c9, t11 CLA Production by Resting Cell

Cell suspensions with different concentrations oxgall and different types of individual bile salts were prepared as described above, and each LA-PBSC without bile salts was used as a control. Cell suspensions were placed in a shaking water bath and incubated at 37 °C for 2 h. All suspensions were collected for analyzing *c*9, *t*11 CLA production as described previously.

### 4.9. Statistical Analysis

Statistical analyses were performed using SPSS 15.0 software (SPSS Inc., Chicago, IL, USA). All experiments were performed in duplicate and repeated three times. Data are presented as the mean ± SD. Significant differences between treatments were tested by ANOVA followed by Tukey's test with a level of significance of *α* = 0.05.

## 5. Conclusions

In conclusion, the inhibitory effect of bile salts on growth and *c*9, *t*11 CLA production of was reversed by Tween 80. The recovery ability was also effective in the presence of 0.1%–0.9% oxgall and 0.3% deconjugated bile salts and conjugated bile salts. This phenomenon is partially attributed to the protection of Tween 80 on leakage of intracellular material. However, all the results in this study were obtained *in vitro*, therefore further *in vivo* experiments will be required. Such work is being performed at our laboratory.
